# HSP70s: From Tumor Transformation to Cancer Therapy

**DOI:** 10.4137/cmo.s475

**Published:** 2008-04-24

**Authors:** Chih-Wen Shu, Chun-Ming Huang

**Affiliations:** 1 Institute of Biotechnology and Department of Life Science, National Tsing Hua University, Hsinchu, Taiwan; 2 Burnham Institute for Medical Research, La Jolla, CA, U.S.A; 3 Department of Medicine, Division of Dermatology, University of California, San Diego; 4 VA San Diego Healthcare Center, San Diego; 5 Moores Cancer Center, University of California, San Diego, CA, U.S.A

## Abstract

Heat shock proteins (HSPs) are a defined set of chaperones for maintaining proper functions of proteins. The HSP70 family, one of the most inducible families in response to stress, protects cells from stress-induced cell death. It has been documented that HSP70s are highly expressed in various types of cancer cells and make the cells resistant to adverse microenvironments, such as hypoxia and glucose starvation, which are common features in malignant progression. Over-expression of HSP70s is thus associated with tumor transformation and eventually results in a decrease of chemotherapy efficacy. Notably, the distribution of HSP70s is deregulated in cancer cells. It has been reported that HSP70s localize distinct organelles or are exported to humoral circulation during cancer development. Either surface or exported HSP70s play danger signals and trigger immune response to destroy the tumor cells. In this review, we lay out recent advances in the HSP70s-mediated cancer diagnosis and therapy. This review would be enlightening for clinical cancer medicine.

## Introduction

In 1962, the chromosome puffs were observed in heat stressed Drosophila larvae and the encoded-genes were identified as heat shock proteins (HSPs).^1^,^2^ HSPs are also known to be mainly transactivated by heat shock factor to chaperone misfolded proteins and rescue cells from adverse environmental stresses, such as PH alteration, ischemia, hypoxia, osmotic and treatment with a number of metals.^3^ According to molecular weight, HSPs are classified into five families including high molecular mass HSP100s, HSP90s, HSP70s, HSP60s and small HSPs.^3^ Moreover, HSP100s function to disaggregate denatured proteins, while small HSPs inhibit aggregation of proteins.^4^–^6^ HSP90s support the folding of premature proteins and play important signal transducers in cell differentiation and proliferation.^7^ HSP70s coordinate with HSP40s to fold nascent polypeptide chains,^8^ while HSP60s, which is so-called chaperonin, and HSP10 are mostly responsible for folding mitochondrial proteins.^9^ Among them, HSP70s is the most important HSPs for protein folding and is highly associated with tumor progression, which may also provide therapeutic strategy of cancer.

## Human Heat Shock Protein 70 Family

In humans, the HSP70 family encompasses multiple distinct genes, which encode a group of highly related proteins: mitochondria resided glucose-regulated protein 75 (GRP75, HSPA9B locus) contains N-terminal mitochondrial localization signal, endoplasmic reticulum (ER) resided glucose-regulated protein 78 (GRP78, HSPA5 locus) contains N-terminal ER localization signal and C-terminal ER retention signal, cytosol/nuclear resided HSC70s (the cognate/constitutive HSP70, HSPA1L, HSPA2 and HSPA8 locus), and also cytosol/nuclear resided but highly inducible and intronless iHSP70s (HSP70/72; HSPA1A, HSPA1B, and HSPA6 loci) (Table 1).^10^ Recent data indicates that some of HSP70s genes are significantly induced in response to distinct stresses in humans. Two of these genes, HSPA1A and HSPA1B, are found as a nearly identical tandem pair in the major histocompatibility complex at 6p21.3 region. HSPA1A and HSPA1B are coded for two almost identical proteins, the major inducible HSP70s in the stressed-cells.^11^,^12^ Another inducible gene of HSP70s, HSPA6, is located on chromosome 1 and is mainly induced by extreme temperature.^13^,^14^ However, the genes of HSC70s are ubiquitously expressed at low levels in most tissues, but show high expression level in specific tissues.^10^,^15^–^17^ On the other hand, specific compartment-localized HSP70s, the genes HSPA5 and HSPA9 are not only constitutively expressed, but also induced under specific stress. HSPA5 is specifically induced in response to ER stress, while HSPA9 is induced by glucose starvation and ionizing radiation.^18^–^20^ It indicates that HSP70s play crucial roles for cell survival under stresses. Additionally, HSP70s are upregulated in a number of cancer cells, suggesting that HSP70s allow the cells to adapt to adverse microenvironments, such as hypoxia and glucose starvation, which are common features in tumor transformation.

## Common Functions of HSP70s: Chaperone and Anti-apoptosis

**Figure 1:**
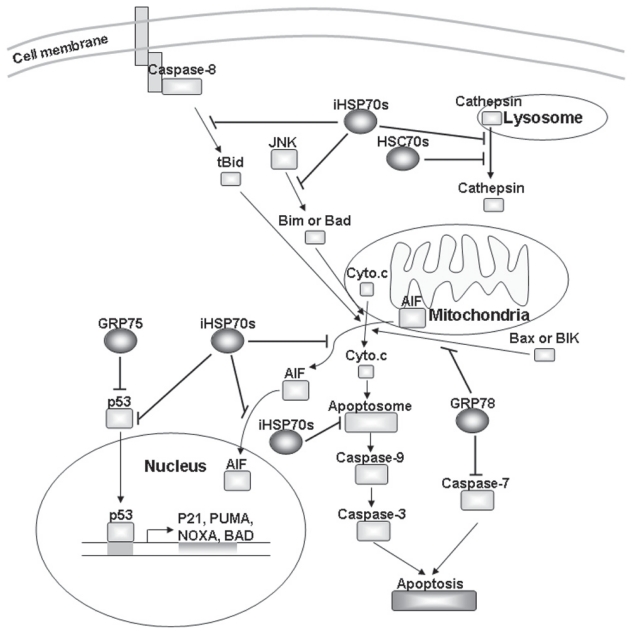
Protective mechanisms of HSP70s in stress-induced cell death HSP70s block the activation of several death factors to attenuate stress-induced cell death. iHSP70s inhibit caspase-8, JNK, p53, AIF and apoptosome formation to protect cell from apoptosis. Moreover, both iHSP70s and HSC70s stabilize lysosomal membrane and decrease cathepsin-dependent cell death. Besides, GRP78 diminishes caspase-dependent apoptosis by inhibiting Bax, BIK and caspase-7 activity. In addition, GRP75 traps p53 in the cytoplasm so that p53 cannot turn on the pro-apoptotic genes.

HSP70s function as chaperones to control protein quality or interact with a number of key regulators to modulate cell proliferation, survival, and death.^21^,^22^ The primary structure of HSP70s consists of around 45 kD ATPase domain and around 25 kD C-terminal peptide binding domain. The C-terminal domain can be further divided into two subdomains: peptide binding subdomain (15kD) and C-terminal subdomain (10 kD).^23^ While ATPase domain stimulates ATP hydrolysis to provide energy for protein folding, EEVD sequence of C-terminal subdomain recruits carboxyl terminus of HSC70-interacting protein with ubiquitin E3-ligase activity (CHIP) to trigger degradation of those malfolded proteins, which cannot be recovered.^24^ HSP70s also associate with co-chaperones including HSP40s and nucleotide exchange factors to efficiently stimulate protein folding. The chaperone activity of HSP70s is also required for blocking apoptosis caused by various stimuli, since deletion of ATPase domain or C-terminal EEVD sequence diminishes the anti-apoptotic effect in response to stress.^25^ However, while iHSP70s serve as safeguards to protect cells from apoptosis in a chaperone-dependent manner, iHSP70s interfere c-Jun N-terminal kinase (JNK) phosphorylation and abrogate JNK-mediated apoptosis in a chaperone independent manner (Fig. 1).^26^

It has been found that the HSP70s prevent stress-induced apoptosis through either mitochondria- dependent or independent pathways (Fig. 1). HSP70s can block activation of the death factors to allow the cells resistance to stress-induced apoptosis.^21^ For instance, iHSP70s attenuate the release of cytochrome c by blocking cleavage of BH3-interacting domain death agonist (Bid) from caspase-8.^27^ iHSP70s coupled with HSP40 prevent Bax mitochondrial translocation.^28^ Moreover, iHSP70s interact with Apaf-1 to forbid its association with procaspase-9.^29^ iHSP70s and HSP70-3 also diminish cell death in caspase- independent pathway by inhibiting apoptosis inducing factor (AIF) and maintaining lysosomal membrane, respectively.^30^,^31^ Furthermore, HSP70-3 siRNA induced p53 expression in HeLa cells, whereas siRNA of iHSP70s did not alter the p53 expression.^32^ Also, PUMA, NOXA and Bcl2 antagonist of cell death (BAD) are critical mediators in apoptosis and transactivated by p53,^33^,^34^ suggesting that HSP70-3 suppresses p53 expression to decrease stress-induced apoptosis.

On the other hand, overexpression of GRP78 suppresses caspase-7/12 activation or stabilizes Raf-1 to maintain mitochondrial permeability, thus diminishing apoptosis in cells treated with ER stress inducers or genotoxic agents.^35^–^38^ Recombinant GRP78 also reduces cytochrome c-induced caspase-3 activation *in vitro.*^39^ Moreover, introduction of antisense GRP78 into cells decreases cell viability during ER stress stimuli.^40^ Knockdown of GRP78 by siRNA arrests cell growth and increases activation of apoptosis regulator Bax or BCL2-interacting killer (BIK) in stress-induced apoptosis.^41^,^42^ Besides, GRP75 interacts with p53 in the cytoplasm to sequester its apoptotic function, thus reducing cell death caused by serum starvation.^43^ Recent studies show that deregulation of HSP70s expression or functions is associated with diseases such as autoimmunity, neurodegenerative diseases, and tumor transformation, particularly in malignant progression.^3^,^44^ It suggests that overexpression of HSP70s in tumor cells confers resistance to adverse microenvironments to promote tumor transformation and decrease chemotherapy efficacy.

## Involvement of HSP70s in Cancer

Overexpression of HSP70s is significantly associated with tumor transformation since HSP70s may block apoptosis to adapt adverse microenvironments as mentioned above or chaperone the mutated proteins of cancer cells, such as mutated p53, which is observed in approximately 50% cancers.^21^,^45^ Moreover, the level of HSP70s is reportedly increased in a variety of tumor cells or tissues as followings (Table 2).

### iHSP70s

Several reports have shown that increased iHsp70s expression is elevated in a variety of malignant tumors, such as colorectal, esophageal and gastric cancer.^46^–^48^ The expression level of HSP70-1 and/or -2 is elicited in either pancreatic cancer cells^49^ or melanoma cell lines.^50^ The importance of these findings is illustrated by the fact that high levels of expression of HSP70-1 and/or HSP70-2 are correlated with hepatoma progression^51^ and increased drug resistance of human breast cancer.^52^,^53^ Overexpression of HSP70-1 and/or HSP70-2 expands the tumor size, metastasis and resistance to chemotherapy.^54^ The polymorphism of HSP70-2 is associated with nasopharyngeal carcinoma^55^ and increases the viability of patients with breast cancer.^56^ Specifically, the cytoplasmic HSP70-1 and/or -2 are more abundant in the colorectal carcinoma than in the normal mucosa.^57^ Also, depletion of the genes HSPA1A and/or HSPA1B by antisense nucleotides in human bladder cancer cell lines BIU-87 sensitizes mitomycin C-based chemotherapy.^58^ Knockdown of the genes HSPA1A and/or HSPA1B by siRNA arrests cell cycle at G2/M phase to diminish cell proliferation of tumorigenic cells including HeLa, MCF-7, PC-3, HuH-7 and gastric cancer SGC-7901 cells, whereas the effect is not observed in nontumorigenic HBL-100 cells.^32^,^59^ However, siRNA of the gene HSPA6 reveals no effect in both HeLa and HBL-100 cells.^32^

### HSC70s

Compared with peripheral blood mononuclear cells (PBMC), HSC70 shows high levels of expression in the epithelial cancer cells including oral cancer (OSC20 and Ca9-22), colon cancer (SW620) and stomach cancer (MKN45).^60^ The gene HSPA2 is elevated in invasive bladder cancer and tissue from patients with breast cancer.^31^,^32^ Nevertheless, HSPA1L shows no correlation with prostate cancer risk.^61^

### GRP75

GRP75 shows induced-expression in many types of brain tumors including meningiomas, neurinomas, pituitary adenomas and metastases.^62^ The mRNA level of GRP75 is upraised in tumor tissues of breast, brain, and colon tumors in the mouse model.^63^ The gene HSPA9 expression in most of tumor or immortalized cell is higher than that in normal cells.^63^ Malignancy of breast cancer is enhanced in GRP75-overexpressed cells.^63^ Besides, elevated expression of GRP75 decreases survival of the patient with colon cancer.^64^

### GRP78

The microenvironment in tumor transformation induces ER stress response to stimulate GRP78 expression. The mRNA and protein level of GRP78 in the tumor tissue are much higher than that in the normal tissue of patients with lung cancer, indicating that the level of GRP78 greatly correlates with malignant progression.^65^ Besides, the expression level of GRP78 is significantly associated with a shorter time of recurrence in patients with breast cancer.^66^ The elevated protein level of GRP78 is also observed in resected tissue from patients with a number of cancer types including liver, lung, prostate, colon, and gastric cancer,^67^–^71^ whereas the mRNA level had no difference in colon cancer, suggesting that posttranscriptional regulation may be involved in the GRP78 expression.^72^ A recent report shows that GRP78 inhibits BIK, a BH3-only proapoptotic protein in ER, or molecularly chaperone Raf-1 to diminish stress-induced apoptosis in human breast cancer MCF-7/BUS cells or non-small cell lung cancer H460 cells.^38^,^73^ Therefore, it indicates that GRP78 could be a treatment marker in chemotherapy.

Heat shock factor 1 (HSF1) and activating transcription factor 6 (ATF6), the transcription factors of iHSP70s and GRP78, respectively, are overexpressed in some certain cancer cells.^74^,^75^ This is probably why the expression level of HSP70s is elevated in tumor cells. Additionally, since the anti-apoptotic and chaperone functions of HSP70s promote tumor transformation, the induction of HSP70s play pivotal roles in carcinogenesis.^66^,^76^ It may also provide diagnostic markers of cancer.

## Distribution of HSP70s in Cancer

Besides the high level expression of HSP70s in tumor cells, HSP70s have also been observed to distribute distinct subcellular localization.^77^–^79^ HSP70s associate with either tumor-specific antigen to localize on the surface or proapoptotic protein to inhibit its function, which may provide a pharmacological strategy in cancer therapy.

### iHSP70s

In addition to nucleus/cytoplasm localization, the minority of HSP70-1 and/or HSP70-2 associated with tumor specific antigens localizes on the cell surface and serves as a danger signal to trigger an immune response in patients with cancer.^80^ Cell surface-localized HSP70-1, HSP70-2 or its derived peptide TKDNNLLGRFELSG (TKD, aa 450–463) interact with CD94 of natural killer (NK) cells to trigger the granzyme B released from NK cells.^81^ The released granzyme B is internalized into tumor cells by interacting with surface-localized HSP70-1 and/or HSP70-2 and initiates apoptosis in a perforin-independent manner.^81^ However, distinct length of TKD-related peptides or TKD-equivalent peptides derived from HSP70-Hom or HSC70 cannot activate NK cells.^82^ Moreover, HSP70-1 and/or HSP70-2 stimulate pro-inflammatory cytokines secretion of antigen presenting cells (APC)s through binding with Toll-like receptors.^83^ Interestingly, HSP70-1 and/or HSP70-2 also exist at high levels in the sera of prostate cancer patients.^84^

### HSC70s

HSC70 has been found on the cell surface as HSP70-1 and/or HSP70-2 and is released to extracellular space in the K562 erythroleukemic cells treated with pro-inflammatory cytokine interferon-gamma (IFN-γ).^78^ Likewise, HSC70 co-localizes with tumor antigens and MHC class I molecules in the exosome released from 2 different types of MHC-mouse cell lines including CT26 (H-2d MHC) and TA3HA (H-2a MHC).^85^ The HSC70-contained exosomes activate dendritic cells and suppress tumor size in the colon cancer-bearing mice.^85^

### GRP75

GRP75 localizes in duplicated centrosome at late G1, S, and G2 phases of the cell cycle, whereas it dissociates with unduplicated centrosome at M phase.^86^ GRP75 is tyrosine phosphorylated at G1 phase in the cells exposed to fibroblast growth factor (FGF) and correlated with FGF-1 induced cell growth.^87^ Moreover, GRP75 interacts with mot-2-binding site (aa 323–337) of p53 to trap p53 in the cytoplasm and inhibit its apoptotic function.^88^

### GRP78

GRP78 is observed in the other sub-cellular compartments except ER. Surface-localized GRP78 is increased in LNCaP prostate cancer cells treated with synthetic androgen R1881.^79^ The peptide sequence LIGRTWNDPSVQQDIKFL(aa 98-115) of surface-localized GRP78 binds to receptor binding domain (RBD) of α2-macroglobulin (α2M*) and serves as a receptor of α2M* to promote cell growth via PI3K/AKT dependent pathway.^89^,^90^ Moreover, GRP78 accumulates in the cytoplasm in hepatocellular carcinoma (HCC).^75^ Besides, recent study shows that autoantibody against GRP78 is increased in sera of the patients with prostate cancer.^90^ Further, the antibody isolated from the patients enhances the proliferation of 1-LN cells expressing GRP78 on the cell surface.^90^ The anti-GRP78 antibody also prevents apoptosis in cells exposed to tumor necrosis factor α^90^.

In addition to distinct intracellular distribution of HSP70s, HSP70s are exported to serum in cancer patients.^91^,^92^ Also, anti-HSP70s antibodies have been determined in some certain cancers.^84^,^90^ Although the function of HSP70s release or autoantibody production needs more studies to examine, it implies that they can be treated as diagnostic or prognostic markers in cancer therapy.

## HSP70s in Cancer Therapy

Since overexpression and dysregulation of HSP70s are involved in tumor transformation, HSP70s play the role of danger signals in patients with cancer. According to the features of HSP70s in cancer, HSP70s—based cancer therapy has been verified in some certain cancers (Table 3).

### iHSP70s

Ex vivo activation of NK cells from patients with metastasized colon and lung cancer by IL-2/TKD peptide treatment has been tested for its tolerability, feasibility, and safety in phase I clinical trial.^93^ The activated NK cells show cytolytic activity to surface HSP70 (HSP70-1 and/or HSP70-2) positive tumor cells in vivo and most patients experienced no negative side effects. Furthermore, the IL-2/TKD peptide- activated NK cells, but not T lymphocyte, which decreased the tumor weight and liver metastasis, thereby increasing the survival rate of pancreatic tumor-bearing mice.^94^ Introduction of antisense cDNA against HSPA1A and/or HSPA1B to nude mice efficiently diminishes the tumor growth of glioblastoma, breast and colon cancer.^53^ Depletion of HSPA1A and/or HSPA1B by siRNA or HSP70s by quercetin, which is an inhibitor of HSF1,^95^ raises the apoptosis of human pancreatic cancer cells including MiaPaCa-2 and Panc-1 in a caspase-dependent manner.^49^ Also, injection of quercetin into the mouse xenografted with MiaPaCa-2 tumor cells significantly attenuates the tumor size.^49^ Likewise, triptolide derived from Triptergium wilfordii blocks HSF1 transcriptional activity without affecting its activation and DNA binding activity, thus diminishing the genes HSPA1A and/or HSPA1B expression and enhances stress-induced apoptosis.^96^ Moreover, cardenolide (UNBS1450), a potent anti-cancer drug for the paclitaxel-and oxaliplatin-resistant tumor, attenuates both mRNA and protein levels of HSP70-1 and/or HSP70-2 in human non-small cell lung cancers partly through suppression of NFAT5/TonEBP, which regulates transcriptional levels of iHSP70s.^97^ It supports the notion that induction of iHSP70s associates with not only malignant progression, but also drug resistance. However, since HSF1 transactivates most of inducible HSPs genes, inhibiting HSF1 not only block iHSP70s expression, but other inducible HSPs, which may appear side effect in the treatment.

### HSC70s

Like HSP70-1 and/or HSP70-2, extracellular HSC70 has been determined to function as a cytokine to activate an immune response leading to tumor suppression.^78^ HSC70-derived peptide EYKGETKSF (aa 106–114) or FDNRMVNHF (aa 233–241) activate cytotoxic T lymphocyte (CTL) in the peripheral blood mononuclear cells of epithelial cancer patients.^60^ Besides, inhibiting the gene HSPA8 expression by siRNA shows anti-proliferative effects in both tumorigenic and non-tumorigenic cells.^32^ Nevertheless, HSP70-3 depletion arrests the cell cycle at G1 phase of HeLa cells, whereas the effect is not observed in normal cells. Depletion of the gene HSPA2 induces expression of macrophage inhibitory cytokine-1, a target of p53, in both p53 dependent and independent pathways to arrest cell cycle at G1 phase.^32^ Suppression of the gene HSPA2 expression also results in cathepsin-dependent cell death via permeabilizing the lysosomal membrane.^31^

### GRP75

Several studies have shown the importance of the interaction between GRP75 and p53 in carcinogenesis.^43^,^88^ Therefore, suppression of the gene HSPA9 expression or interference with interaction would be a therapeutic strategy against cancer. Indeed, knockdown of the gene HSPA9 by ribozyme or siRNA reduces cell growth and viability of human cancer cells.^98^,^99^ Disrupting the interaction between GRP75 and p53 by the potent anti-cancer drug, MKT-077,^100^ or GRP75 binding peptide, activates endogenous p53, thus preventing cell growth of osteosarcoma and breast carcinoma cells.^88^

### GRP78

Since GRP78 associates with tumor transformation and cancer therapy, it provides a good therapeutic target for several potent anti-cancer drugs. For instance, suppression of HSPA5 by siRNA increases sensitivity of tumor cells to etoposide in mice xenografted with human breast cancer.^101^ Moreover, Melanoma differentiation-associated gene-7/interleukin-24 (MDA-7/IL-24) and its derived peptide M4 interact with GRP78 in ER lumen to trigger ER stress response, which in turn promotes apoptosis of cancer cells.^102^ Injection of MDA-7/IL-24 recombinant adenoviruses (Ad. Mda-7) into breast tumor-bearing mice results in a decrease of tumor volume.^102^ In phase I clinical trial of Ad. Mda-7 in patients with melanoma, it has been determined to be safe and efficient in inducing apoptosis of melanoma.^103^ Additionally, SubAB, a toxin from the highly virulent strain of *Escherichia coli,* cleaves GRP78 at Leu 416 site to destroy its function, which may provide a therapeutic application.^104^ On the other hand, surface-localized GRP78 could be utilized as a carrier of anti-cancer drugs. The cyclic 13-mer oligopeptide, Pep42, interacts with surface-localized GRP78 and internalizes to lysosome of tumor cells in a clathrin-dependent manner.^105^ Pep42 also targets tumor tissue in the xenograft mice with melanoma cells. Furthermore, Pep42 conjugated with the apoptosis-inducing oligopeptide (AIO) or photosensitizer decreases viability of tumor cells, such as A549 and HepG2 cells; whereas the effect is not observed in normal cells.^105^ Likewise, the GRP78 binding peptides, WIFPWIQL or WDLAWMFRLPVG, fused with AIO decreases the tumor size in the xenograft mice with prostate and breast cancers.^106^

While some of the surface-localized HSP70s (HSP70-1, HSP70-2, and HSC70) serve as danger signals in cancer patients, surface-localized GRP78 serves as a receptor of α2M* to promote cancer cell growth. Inhibiting HSP70s genes expression by siRNA or inhibitors might also diminish the danger signal from the surface-localized HSP70. Therefore, HSP70s-based cancer therapy may depend on the specificity of induced or surface-localized HSP70s in the patient.

## Conclusion and Perspective

While HSP70s play pivotal roles for general peptide folding to maintain normal physiological function of cells, dysregulation of expressional and spatial control of HSP70s causes tumor transformation and decreases chemotherapy efficacy. Therefore, comprehension of molecular mechanisms regarding gene regulation and protein function of HSP70s might provide a paradigm for cancer therapy. Although plenty of studies about HSP70s-based cancer therapy have been determined, most of the studies are still in cell or preclinical models. The efficacy of HSP70s-based cancer therapy needs to be further verified in the clinical trial. On the other hand, since overexpressed HSP70s demonstrate variable localization as mentioned above and some of HSP70s reveal posttranslational modification in tumor cells,^107^ the relevance between post-translational modification and distribution of HSP70s remains unclear. To determine the detailed mechanism of HSP70s in tumor progression, HSP70s-based chaperosome using a proteomics approach would be informative in the regulation of HSP70s in cancers, which in turn would shed light on the cancer therapy.

## Figures and Tables

**Table 1:** Classification of human heat shock protein 70 family.

Protein	Gene locus symbol	Intracellular localization	Alternative name	PI/MW (kDa)	Inducibility	Reference
**iHSP70s**						
HSP70-1	HSPA1A	Nu/Cyto/Lyso	HSP72, Hsp70, Hsp70i, Hsp70-1a	5.48/70.0	Yes	11, 12
HSP70-2	HSPA1B	Nu/Cyto/Lyso	HSP72, Hsp70, Hsp70-1b	5.48/70.0	Yes	11, 12
HSP70B′	HSPA6	Nu/Cyto	Hsp7070-6	5.76/70.4	Yes	13, 14
**HSC70s**						
HSC70	HSPA8	Nu/Cyto	Hsp70-8, HSP73	5.81/71.0	No	15
HSP70-Hom	HSPA1L	Nu/Cyto	Hsp70-1l, Hsp70t	5.48/70.0	No	16
HSP70-3	HSPA2	Nu/Cyto	Hsp70-2, Hsp70-2b	5.07/72.3	No	17
**GRP70s**						
GRP75	HSPA9	Mito	Mortalin, mtHsp75, Hsp70-9, PBP74	5.37/70.9	Yes	19, 20
GRP78	HSPA5	ER	Bip, Hsp70-5	6.03/73.7	Yes	18

**Abbreviations:** Nu: nucleus; Cyto: cytoplasm; Mito: mitochondria; ER: endoplasmic reticulum; PBP: peptide binding protein; Bip: Immunoglobulin heavy chain-binding protein homolog.

**Table 2:** Association of HSP70s in cancers.

Protein	Findings	Cancer type	Reference
iHSP70s	HSP70-1 and/or -2 expression are increased in the cancer	Breast cancer	52
		Colon cancer	48
		Gastric cancer	46
		Melanoma	50
		Tumorigenic cells (HeLa, MCF-7, PC-3, HuH-7, SGC-7901)	32, 59
	HSP70-2 polymorphism associates with the cancer	Nasopharynx cancer	55
	HSP70-1 and/or -2 decrease chemotherapy efficiency	Bladder cancer (BIU-87)	58
HSC70s	HSP70-3 expression is increased in the cancer and reduce cell death	Bladder cancer	31
	HSP70-3 regulates cell proliferation	Cervix cancer (HeLa)	32
GRP75	GRP75 expression is elevated in the cancer	Brain cancer	62
		Breast cancer	63
		Colon cancer	63
	Elevated GRP75 in cancer cells decreases survival of the patient	Colon cancer	64
GRP78	GRP78 expression is increased in the cancer	Liver cancer	75
		Lung cancer	72
		Colon cancer	70
		Gastric cancer	68, 71
	GRP78 shortens the time of recurrence	Breast cancer	66

**Table 3:** Potential HSP70s-based cancer therapy.

Protein	Mechanism of action	Therapy	Reference
iHSP70s	Activate NK cells against cancer cells with surface iHSP70s	IL-2/TKD peptide (immunotherapy)	93 94
	Inhibit iHSP70s expression	Antisense iHSP70s cDNA	53
		siRNA	49
		Quercetin	49
		Cardenolide (UNBS1450)	97
		Triptolide	96
HSC70s	Activate CTL	HSC70-derived peptide (immunotherapy)	78
	Inhibit HSC70s expression	siRNA	32
GRP75	Inhibit GRP75 expression	ribozyme	98
		siRNA	99
	Disrupt interaction between GRP75 and p53	MKT-077	100
		GRP75 binding peptide	88
GRP78	Inhibit gene expression	siRNA	71, 101
	The binding peptide conjugated with cytotoxic peptide kill the cancer cells with surface localized-GRP78	GRP78 binding peptides	105, 106
	Cleave GRP78 at Leu 416	SubAB	104
	Block GRP78 in the ER	MDA-7/IL-24 recombinant adenoviruses	102
